# Staphylococcus lugdunensis Native Valve Endocarditis in a Patient With Confirmed COVID-19: A Cautionary Tale of an Old Disease Not to Be Forgotten in the Pandemic Era

**DOI:** 10.7759/cureus.17724

**Published:** 2021-09-05

**Authors:** Ioannis Mouzakitis, Zoi Kleinaki, Theodora Petropoulou, Erato Alexiou, Alexandros Stefanidis

**Affiliations:** 1 Internal Medicine, General Hospital of Nikea, Athens, GRC; 2 Cardiology, General Hospital of Nikea, Athens, GRC

**Keywords:** covid-19, sars-cov-2, infective endocarditis, coagulase-negative staphylococci, trans thoracic echocardiography

## Abstract

The coronavirus disease 2019 (COVID-19) pandemic has radically altered priorities and planning in the global medical community with continuously increasing numbers of infected patients. Cardiovascular disease accounts for the most common comorbidity encountered in patients with COVID-19. Infective endocarditis (IE) represents a grave medical condition as it may compromise circulatory homeostasis, renal function, and lead to embolic complications. COVID-19 also causes coagulopathy-associated complications. In this report we describe the case of a patient presenting to the accident and emergency department with fever and chills. COVID-19 was confirmed and further workup revealed concomitant severe native valve IE with *Staphylococcus lugdunensis*. Medical community worldwide should remain alert and continue working towards early recognition of these intricate interactions.

## Introduction

The coronavirus disease 2019 (COVID-19) pandemic has radically altered priorities and planning in the global medical community. COVID-19 has affected more than 218,800,000 patients worldwide to date, resulting in more than 4,500,000 deaths [1]. Diagnosing and managing a patient with comorbidities presenting with fever has become challenging, as severe acute respiratory syndrome coronavirus 2 (SARS-CoV-2) infection should be ruled out in the current setting. That realization notwithstanding, diagnosis of COVID‐19 does not imply the exclusion of other diseases.

Cardiovascular disease is the most common comorbidity found in patients with COVID-19 [2]. As a result, clinical presentation of COVID-19 can often be misleading, as it shares many characteristics with an acute or chronic decompensated cardiovascular event [3]. Infective endocarditis (IE) represents a grave medical condition that may compromise circulatory homeostasis and renal function and lead to complications such as embolic phenomena and abscesses in the central nervous system, spleen, kidneys, or skin [4]. On the other hand, COVID-19 also causes coagulopathy-associated complications and damage to many organs and systems [5]. The clinical manifestations of IE and COVID-19 are challenging and both diseases may present at first with non-specific symptoms such as fever, chills, dyspnea, fatigue, cough, and myalgia. In this report, we describe the case of a patient with confirmed COVID-19 and concomitant severe native valve IE.

## Case presentation

An 83-year-old female patient was referred to the accident and emergency department of our hospital in mid-October 2020, with a five-day history of fever and chills. No dyspnea, diarrhea, cough or sore throat were initially reported by the patient. The patient had a past medical history of coronary heart disease, arterial hypertension, and diabetes mellitus. An initial reverse transcription-polymerase chain reaction (RT-PCR) test of a nasopharyngeal swab had been negative for SARS-CoV-2 RNA. Family members and relatives of the patient were also tested negative for SARS-CoV-2 with RT-PCR. Thus, she was consequently admitted to the internal medicine department.

The patient was alert but ill-appearing on general inspection. Vital signs included: arterial blood pressure of 140/70 mmHg, heart rate ranging from 90 to 100 beats per minute, body temperature of 37.6°C, and respiratory rate of approximately 20 breaths per minute. On heart auscultation, a murmur was detected in the aortic region (second right intercostal space). The pulmonary examination was clear to auscultation. Abdomen was soft, non-tender, with normal bowel sounds. A chest CT examination was performed, as per the hospital’s standard procedure in every patient presenting with fever since the pandemic was declared, with unremarkable findings. Initial laboratory tests reflected leukocytosis, relative lymphopenia, slightly elevated high sensitivity troponin I (169.4 ng/ml), kidney function alteration (estimated glomerular filtration rate-eGFR 32.7 ml/min), elevated C-reactive protein, and mild hyponatremia. Procalcitonin was positive at 0.7 ng/mL with a cut-off value of 0.4. Arterial blood gas analysis showed no noteworthy abnormality on admission with pH 7.44, PaCO2 30 mmHg, PaO2 94 mmHg, HCO3− 20.4 mmol/l, oxygen saturation of 98%, with no oxygen supplementation required. The patient was again febrile, at 38.6oC, 12 hours after admission. Two sets of blood cultures were obtained with a difference between the two venipunctures of 30 minutes. At the same time, urine culture was also sent to the microbiology department.

In 48 hours from admission, the patient developed shortness of breath, requiring oxygen supplementation, congestive heart failure, and acute renal injury. A second confirmatory RT-PCR for SARS-CoV-2 RNA was then requested. This test was positive and, in accordance with hospital protocols, the patient had to be transferred to the designated COVID-only ward. The patient was started on a regimen of dexamethasone and remdesivir intravenously, as per current National Institutes of Health (NIH) guidelines [6]. The blood cultures obtained after admission isolated a coagulase-negative, Gram-positive bacterial microorganism, *Staphylococcus lugdunensis.* Pending sensitivity analysis, linezolid was initially administered intravenously. The report was available 48 hours later and linezolid was stopped. Vancomycin and gentamycin were accordingly added to her treatment plan. Vancomycin and gentamycin serum levels were monitored to optimize effectiveness. A follow-up imaging of the lungs revealed newly formed bilateral ground-glass opacities and patchy consolidation areas. There was also evidence of a bilateral pleural effusion, treated with loop diuretics. An echocardiogram was initially planned but then postponed due to the patient’s worsening clinical status and her being isolated in the COVID-only ward, taking into consideration the risk of possible contamination of healthcare personnel.

Fever subsided gradually over the next 10 days and, after testing twice negative in new RT-PCRs of nasopharyngeal swab samples, ensuring minimal viral shedding, the patient returned to the internal medicine department for further treatment. On examination, apart from the aforementioned clinical features, painless, non-pruritic petechiae-like, and papulovesicular rash lesions could now be observed on the patient’s upper and lower limbs, trunk while eyelids, palms, and soles were spared.

**Figure 1:**
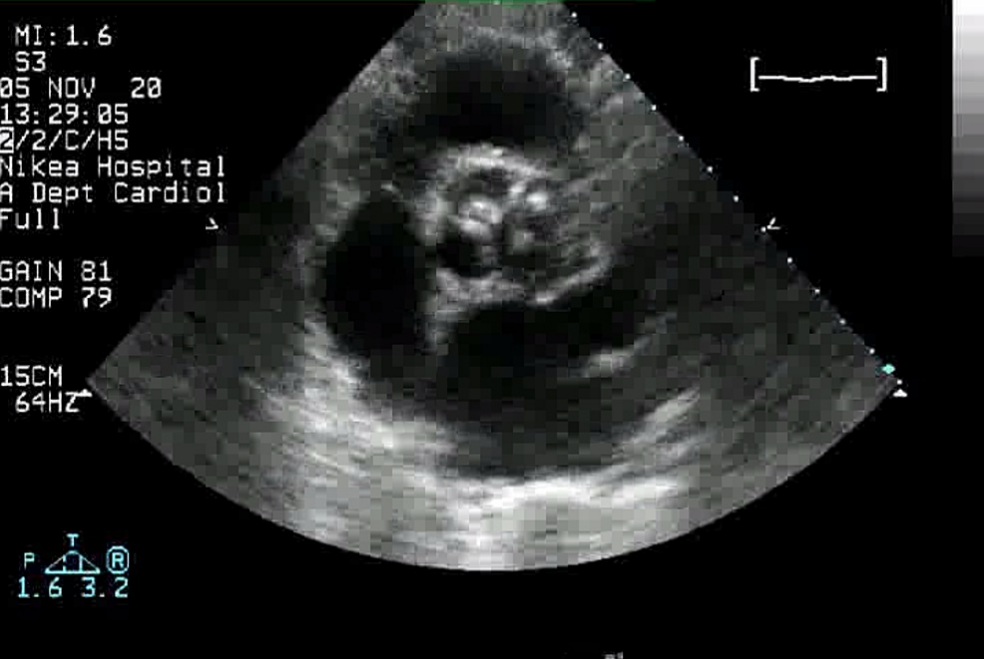
TTE depicting the abscess in the patient’s native aortic valve and surrounding tissue. TTE: transthoracic echocardiogram

**Figure 2:**
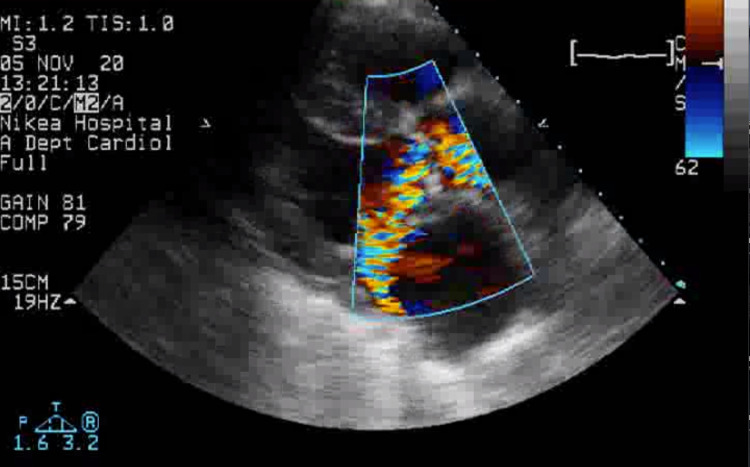
Doppler mode in TTE of the patient’s native aortic valve. TTE: transthoracic echocardiography)

A transthoracic echocardiogram (TTE) was then requested and obtained. Imaging was remarkable for the presence of an abscess in the patient’s native aortic valve and surrounding tissue, which extended almost to the neighboring mitral valve, causing severe insufficiency of both (Figure 1 and Figure 2). The aforementioned findings were consistent with a diagnosis of complicated native-valve bacterial endocarditis. Valve replacement surgery was considered, but the patient’s status at that time was prohibitive. The patient received antibiotic and supportive therapy, but her condition continued to deteriorate, and she eventually passed away on hospital day 20.

## Discussion

In the current COVID-19 pandemic, symptoms related to IE might be incorrectly attributed to a diagnosis of SARS-CoV-2 infection. Observational studies conducted in Spain and Belgium report a 34% and 33% decrease in the absolute number of IE definitive diagnoses respectively when comparing 2020 with 2019, indicating a substantial risk of underdiagnosis [7-8]. Conversely, COVID-19 itself might represent a risk factor for IE, increasing the probability of a concomitant presentation. Due to the inflammatory response induced by this viral agent, the likelihood of IE, even in patients with no other prior predisposing factors, may be increased [9-10]. The damage to a valve structure by the cytokine storm with the systemic inflammation and the hypercoagulable state induced by COVID-19 infection may also contribute as preceding risk factors [9]. Moreover, the upregulated coagulation state by recent COVID-19 infection further helps microorganisms to encase in a platelet/fibrin matrix on the heart valve structure [9-10]. In the current case, differential diagnosis was even more challenging owning to the dermatological manifestations observed. Petechiae-like and papulovesicular rash lesions could be consistent with either vasculitis or IE or microemboli related to the COVID-19 infection [11].

It should also be noted that patients might avoid medical care during the pandemic and hospital resources might be relocated according to the needs [8]. Even though transesophageal echocardiography (TEE) is a very sensitive examination and the cornerstone for the diagnosis of IE, current recommendations of European Association of Cardiovascular Imaging (EACVI) suggest that its use should be restricted due to the high risk of contamination of the operators [12]. Substitution of TEE with TTE might be an explanation for the worse prognosis of patients diagnosed with IE during the pandemic, as these patients potentially have larger vegetations, possibly an indirect indicator of severity. This has an impact on the statistics regarding IE diagnosed during the pandemic, with fewer cases recorded, recognized, and treated promptly, leading to an increased percentage of unfavorable outcomes [7,13].

*Staphylococcus lugdunensis* is a normal skin commensal that preferentially colonizes the perineal region, but as a pathogen, it can cause destructive IE, with ventricular septal defect and destruction of the aortic and mitral valves [14,15]. In parallel with other coagulase-negative Staphylococci (CoNS), it has the ability to adhere and produce biofilm, which enables growth on bioprosthetic materials as well as native tissues [14]. Though CoNS, in general, can be considered a contaminant, *S. lugdunensis* should be regarded as a pathogen [16]. *S. lugdunensis* causes a spectrum of diseases including skin and soft tissue infection, bacteremia, aggressive IE with significant mortality rates, urinary tract infections, peritonitis, prosthetic joint infections, osteomyelitis, arthritis, and catheter-related infection [16]. These infections commonly affect the middle-aged to elderly patient populations, with greater prevalence in females, with concurrent comorbidities including chronic immunosuppressive therapy or diabetes mellitus, as was the case of our patient [16-17]. Surgical treatment is needed for most patients with *S. lugdunensis* IE in order to improve the mortality rate [17]. To our knowledge, this is the first study describing a co-infection of COVID-19 with *S. lugdunensis* IE. 

The overall facts and assessment of this clinical presentation suggest that a simultaneous occurrence of both COVID-19 pneumonia and IE is possible and should not be excluded once a presumptive diagnosis has not been reached. Bibliographical data and a number of case reports seem to support that assessment [18-20].

## Conclusions

IE remains an extremely severe disease, with a high mortality rate. Timely diagnosis and appropriate treatment are indispensable if a negative outcome is to be avoided. During the COVID-19 pandemic, however, the global emergency tends to overwhelm healthcare and dominate physicians' attention, while alternative diagnoses tend to be overlooked. More complex clinical patterns and co-infections will continue to emerge as COVID-19 becomes an integral part of the overall medical landscape. As the crisis gradually subsides, the medical community worldwide should remain alert and continue working towards early recognition of these intricate interactions, especially when frail, elderly, or immunocompromised patients are concerned. 
